# Macrophage P2X7 Receptor Function Is Reduced during Schistosomiasis: Putative Role of TGF-***β***1

**DOI:** 10.1155/2014/134974

**Published:** 2014-08-24

**Authors:** Suellen D'arc Santos Oliveira, Hayandra Ferreira Nanini, Luiz Eduardo Baggio Savio, Mariana Caldas Waghabi, Claudia Lucia Martins Silva, Robson Coutinho-Silva

**Affiliations:** ^1^Laboratory of Immunophysiology, Institute of Biophysics Carlos Chagas Filho, Federal University of Rio de Janeiro, Rio de Janeiro, Brazil; ^2^Laboratory of Molecular and Biochemical Pharmacology, Institute of Biomedical Sciences, Federal University of Rio de Janeiro, 21941-599 Rio de Janeiro, RJ, Brazil; ^3^National Institute of Translational Research in Health and Environment in the Amazon Region, Rio de Janeiro, Brazil; ^4^Laboratory for Functional Genomics and Bioinformatics, Oswaldo Cruz Institute, FIOCRUZ, Rio de Janeiro, Brazil; ^5^Instituto de Biofisica Carlos Chagas Filho, UFRJ, Edifício do Centro de Ciências da Saúde, Bloco G., Avenida Carlos Chagas Filho 373, Cidade Universitária, Ilha do Fundão, 21941-902 Rio de Janeiro, RJ, Brazil

## Abstract

Schistosomiasis is a chronic inflammatory disease whose macrophages are involved in immunopathology modulation. Although P2X7 receptor signaling plays an important role in inflammatory responses mediated by macrophages, no reports have examined the role of P2X7 receptors in macrophage function during schistosomiasis. Thus, we evaluated P2X7 receptor function in peritoneal macrophages during schistosomiasis using an ATP-induced permeabilization assay and measurements of the intracellular Ca^2+^ concentration. ATP treatment induced significantly less permeabilization in macrophages from *S. mansoni*-infected mice than in control cells from uninfected animals. Furthermore, P2X7-mediated increases in intracellular Ca^2+^ levels were also reduced in macrophages from infected mice. TGF-*β*1 levels were increased in the peritoneal cavity of infected animals, and pretreatment of control macrophages with TGF-*β*1 reduced ATP-induced permeabilization, mimicking the effect of *S. mansoni* infection. Western blot and qRT-PCR data showed no difference in P2X7 protein and mRNA between uninfected, infected, and TGF-*β*1-treated groups. However, immunofluorescence analysis revealed reduced cell surface localization of P2X7 receptors in macrophages from infected and TGF-*β*1-treated mice compared to controls. Therefore, our data suggest that schistosomiasis reduces peritoneal macrophage P2X7 receptor signaling. This effect is likely due to the fact that infected mice have increased levels of TGF-*β*1, which reduces P2X7 receptor cell surface expression.

## 1. Introduction

Macrophages are plastic phagocytic cells that participate in innate and adaptive immunity, with important roles in the response against extra- and intracellular parasites, as well as in tissue homeostasis [1, 2]. These cells have complex functions during acute and chronic inflammation. In inflammatory foci, macrophages contribute to mounting specific immunological responses for host defense, and the plasticity of macrophage phenotypes is modulated by the cytokine profile [1].

Tissue damage during inflammation increases the extracellular levels of otherwise intracellular molecules, resulting in damage-associated molecular patterns (DAMPs) recognized by the immune system [3–5]. Extracellular nucleotides, such as the ATP released by dying cells and by activated immune cells during inflammation, are important danger signals involved in immune response [6], participating in both paracrine [7] and autocrine [8] signaling pathways. In inflammatory processes, high extracellular ATP levels generated by tissue damage or secretion are recognized by the immune system as a danger signal, activating purinergic P2 receptors that contribute to a proinflammatory response [9].

Purinergic P2 receptors can be classified as ionotropic ATP-gated (P2X) or G-protein coupled metabotropic (P2Y) [10]. Extracellular ATP (eATP) induces macrophage-mediated immune responses mainly through the activation of P2X7 receptors [11]. These receptors have a ubiquitous distribution, although the highest levels of receptor expression are observed in immune cells of monocyte/macrophage origin [12]. P2X7 receptor activation induces a myriad of intracellular events, including the production of nitric oxide (NO) and of reactive oxygen species (ROS) and the activation of phospholipase-D (PLD). These events are important for intracellular parasite killing, for the release of proinflammatory cytokines (such as IL-1*β*), and for inducing apoptosis [13, 14].

The expression and function of P2X7 receptors are regulated by both pro- and anti-inflammatory stimuli [15]. Initially, P2X7 receptor activation in macrophages opens a plasma membrane cation channel that allows a substantial efflux of K^+^, and influx of Ca^2+^, with later formation of pores permeable to large molecules [16, 17]. P2X receptor activation may also induce caspase-1 activation, interleukin IL-1*β* release, apoptosis, phagolysosomal fusion, and the elimination of intracellular pathogens [14, 18, 19]. In murine macrophages, the mature IL-1*β* secretion by the activation of P2X7 receptors is reduced by the incubation in a free-Ca^2+^ buffer [20]. Thus, murine macrophage release of mature IL-1*β* depends on intracellular Ca^2+^, confirming that Ca^2+^ signaling is essential for this P2X7 receptor function.

Schistosomiasis is a chronic inflammatory disease caused by* Schistosoma mansoni* and it represents the second most common tropical parasitic disease related to socioeconomical factors. While migrating through the vascular system of infected mammalian hosts, parasites evolve from the schistosomula migratory form into adult worms, causing endothelial cell activation, immunological responses, and tissue damage. The disease starts with a Th1-type immune response that gradually changes to a Th2 profile [21–24]. Previous data suggest that the Th2 “stage” begins four to six weeks after infection and is related to egg deposition by adult worms.

Macrophages play important roles in both phases of schistosomiasis [24]. In the early phase of the disease, macrophages act as immune system effectors cells killing schistosomula and promoting tissue repair [24]. After egg deposition, the immune response switches to a Th2 profile that is involved in the formation of liver and colonic granulomas and fibrosis. The eggs are laid in mesenteric microcirculation and they may reach peritoneal cavity where the granulomas formed contain mainly macrophages [25]. Although granulomas are important to limit egg-derived potential cytotoxic products, macrophages isolated, for example, from hepatic granulomas, produce lipids mediators and free radicals that are potentially destructive to host tissues [26]. Among cytokines involved in schistosomiasis, transforming growth factor-*β*1 (TGF-*β*1) is of particular interest, since high levels of this cytokine are released by peripheral blood mononuclear cells (PBMCs) from* S. mansoni*-infected mice [27]. TGF-*β* has an important role in immune modulation later during infection, limiting liver inflammation and favoring host survival [27, 28].

Recently, Bhardwaj and Skelly [29] showed that* S. mansoni* expresses P2X7 receptors-like molecules and also enzymes responsible for the clearance of extracellular ATP (ectonucleotidases), suggesting that purinergic signaling is conserved in these parasites and is important for the host-parasite interplay. However, the impact of the chronic inflammation trigged by schistosomiasis on macrophage P2X7 receptor function remains unknown.

Here, we evaluated P2X7 receptor function and expression in macrophage from* S. mansoni*-infected mice. Our data show that peritoneal macrophage P2X7 receptor function is attenuated during schistosomiasis and that this is associated with high peritoneal levels of TGF-*β*1, the important inflammatory mediator present in the chronic phase of the disease. We also show that TGF-*β*1 downregulates P2X function* in vitro*, mimicking the effect of* S. mansoni* infection.

## 2. Materials and Methods

### 2.1. Reagents and Antibodies

The following primary antibodies were used in this work: rabbit polyclonal anti-P2X7 receptors (APR-004 and APR-008; Alomone Labs, Israel); mouse monoclonal anti-*β*-actin (Santa Cruz Biotechnology, USA); rat anti-F4/80 (Biolegend, USA); rat anti-F4/80 FITC (AbD Serotec, USA). ATP, 3′-O-(4-benzoyl)-ATP (BzATP), PMSF, sodium orthovanadate, aprotinin, leupeptin, BSA, ionomycin, and EGTA were from Sigma Chemical Co. (USA). Fura-2-AM was from Molecular Probes (USA) and A740003 was from Tocris (USA). RPMI, foetal bovine serum (FBS), and penicillin/streptomycin solutions were from GIBCO BRL (USA). TGF-*β*1 was from R&D Systems (Minneapolis, MN, USA). Stock solutions were prepared in DMSO (2.5 mM Fura-2-AM and 10 mM A740003), water (10 mM BzATP), RPMI (5 ng/mL TGF-*β*1), or a buffered physiological saline solution (10 mM ATP). The highest concentration of solvent used was 0.1% (v/v). All other reagents used were of analytical grade.

### 2.2. Mice

*Swiss*, C57BL/6 (wild type), and P2X7 receptor knockout (P2X7 KO) male mice were used in all procedures.* Swiss* and C57BL/6 mice were obtained from the animal facility of the Paulo de Goes Microbiology Institute (Federal University of Rio de Janeiro, Rio de Janeiro, Brazil). P2X7 KO mice (originally from The Jackson Laboratory, USA, stock number 005576) were maintained in the transgenic animal house of the Federal University of Rio de Janeiro. All mice were kept under a light/dark cycle of 12/12 h and with access to water and food ad libitum.

All experiments were conducted in compliance with the ethical standards of our institution (Ethics Committee of the Federal University of Rio de Janeiro; approved under the licenses DFBC-ICB-011 and IBCCF 154) and following both the guidelines of the National Council on Experimental Animal Control (CONCEA, Brazil) and the Committee of Care and Use of Laboratory Animals (National Research Council, United States). All efforts were made to minimize both animals suffering and the number of animals used, on the basis of valid statistical evaluation.

### 2.3. Parasite and Infection

In this work, we used the BH strain of* S. mansoni*, obtained from infected* Biomphalaria glabrata* snails.* Swiss*, C57BL/6, and P2X7 KO mice (7-10 days old) were infected percutaneously with approximately 80 cercariae from both genders, for 8 min, as previously described [30]. Animals were used in experiments at least 45 days after infection (dpi) to allow for full establishment of the infection. Age-matched (60 to 80 days old) uninfected* Swiss*, C57BL/6, and P2X7 KO mice were used as controls. C57BL/6 and P2X7 KO mice survival was evaluated during infection.

### 2.4. Peritoneal Macrophages

To obtain mouse peritoneal macrophages, animals were anesthetized, sacrificed by cervical dislocation, and cleaned with 70% ethanol. The peritoneal cavity was washed with 5 mL of sterile phosphate buffered saline (PBS: 137 mM NaCl, 8.1 mM Na_2_HPO_4_, 1.5 mM NaH_2_PO_4_, and 2.7 mM KCl, pH 7.4), and the peritoneal exudate was collected and centrifuged at 350 ×g, for 5 min, at 4°C. The pellet was resuspended in 1 mL of PBS and used to perform total leukocyte counts (cell viability, as estimated by Trypan blue exclusion, was always ≥95%). For macrophages culture, cells were resuspended in 1 mL of RPMI-1640 medium containing 2 g/L sodium bicarbonate, 1 mM L-glutamine, 100 U/mL penicillin, and 100 *μ*g/mL streptomycin and plated in 35 mm Petri dishes or on 6-well plates. After 1 h of incubation at 37°C with 5% CO_2_, nonadherent cells were removed by vigorous washing, and RPMI-1640 medium with 10% heat-inactivated FBS was added to the cultures, which were kept at 37°C with 5% CO_2_ for 24 h until further use.

### 2.5. ATP-Induced Permeabilization Assay

To investigate macrophage responses to ATP, freshly harvested peritoneal macrophages (10^5^ cells) from* Swiss* (uninfected or* S. mansoni*-infected) or C57BL/6 (wild type or P2X7 KO) mice were treated with 0.1, 0.5, or 1 mM ATP and 2.5 *μ*M ethidium bromide (EB) in PBS (15 min; 37°C). EB was used as a tracer for P2X7 receptor activation (i.e., cell permeabilization) triggered by ATP. After incubation with ATP and EB, F4/80 positive cells were analyzed by flow cytometry (10,000 events/sample) using a FACScan system (BD Pharmingen, USA). Flow cytometry data analysis was performed using the WINMDI 2.9 software. In each histogram, a marker was added based on a gate of the F4/80 positive macrophages population, which limited the basal fluorescence. The specific ATP-induced permeabilization threshold was defined by comparison with the “baseline” profile of control cells not treated with ATP (i.e., incubated with EB only). The specific permeabilization is the percentage of EB positive cells after ATP stimulation in the F4/80 positive gated cells.

Alternatively, peritoneal macrophages were plated on 24-well plates (2 × 10^5^ cells/well) and, after 24 h, treated with 5 ng/mL TGF-*β*1 or vehicle (RPMI), for 24 h, at 37°C and in 5% CO_2_. Macrophages were washed in PBS (pH 7.4) and then incubated with 1 mM ATP and 5 *μ*M EB in buffer (145 mM NaCl, 5 mM KCl, 1 mM MgCl_2_, 1 mM CaCl, and 10 mM HEPES, pH 7.4) for 15 min at 37°C to evaluate the P2X7 receptor activation. The ratio between permeabilized (i.e., EB positive) and total cells was determined by direct counting of cells in five randomly chosen fields, using an Axiovert 100 microscope (Karl Zeiss, Oberkochen, Germany) equipped with an Olympus digital camera (Olympus American Inc., PA, USA). The specific ATP-induced permeabilization value (in %) was calculated by subtracting from the total % of permeabilized cells a “baseline” value corresponding to the % of permeabilized cells in control samples not treated with ATP (i.e., incubated with EB only).

### 2.6. Measurement of [Ca^2+^]_*i*_ in Macrophages

Measurements of the intracellular Ca^2+^ concentration ([Ca^2+^]_*i*_) using Fura-2-AM were performed as previously described [31]. Briefly, macrophages were plated on glass bottom plates (MatTek, USA), at a density of 1 × 10^5^ cells/plate, and maintained for 24 h at 37°C, with 5% CO_2_. Then, the medium was removed and the cells were incubated for 40 min with 2.5 *μ*M Fura-2-AM at room temperature. Cells were subjected to alternate cycles of illumination with 340 nm and 380 nm excitation wavelengths, and the emission was measured at 500 nm. Cells were washed twice with buffered physiological solution (in mM: NaCl 140, KCl 5, MgCl_2_ 1, CaCl_2_ 2, glucose 5, and HEPES 5, pH 7.4) and then stimulated with 100 *μ*M 3′-O-(4-benzoyl)-ATP (BzATP) (at 37°C) in the absence or in the presence of 50 nM of the P2X7 receptor antagonist A740003 (preincubated for 45 min, at 37°C). After the maximum effect had been achieved, cells were treated with Ca^2+^ ionophore (10 *μ*M ionomycin) and then with 6 mM EGTA to determine the molar concentration of Ca^2+^ according to the Grynkiewicz equation [32].

### 2.7. Determination of TGF-*β*1 Levels

The estimation of TGF-*β*1 levels in mouse peritoneal washes was performed using an ELISA immunoassay kit (Peprotech, Rocky Hill, NJ, USA), according to manufacturer's instructions. For peritoneal wash collection, mice were euthanized with CO_2_ and the peritoneal cavity was washed with 1 mL of sterile PBS. Approximately 60% of the initial volume of PBS was recovered and centrifuged at 350 g for 5 min, at 4°C. Supernatants were collected and stored in liquid N_2_ until further use (to determine TGF-*β*1 content).

### 2.8. Confocal Microscopy

Peritoneal macrophages from control and infected mice (10^5^ cells/well) were treated with 5 ng/mL TGF-*β* for 24 h, fixed with 4% paraformaldehyde and 4% sucrose for 10 min in PBS, washed, and incubated for 30 min with 50 mM ammonium chloride (pH 8.0). After three washes in PBS, samples were blocked in PBS with 10% FBS and 0.1% BSA for 30 min, washed twice in PBS, and incubated overnight with a rabbit polyclonal antibody that recognizes an extracellular epitope of P2X7 receptors (APR-008; Alomone Labs, Israel; Peptide KKGWMDPQSKGIQTGRC, corresponding to amino acid residues 136–152 of mouse P2X7 receptor; extracellular loop) diluted to 1 : 400 in PBS and 0.1% BSA. Then, cells were washed and incubated with a rat anti-F4/80 antibody diluted to 1 : 50 in PBS and 0.1% BSA for 4 h, at 4°C. Samples were then labelled with secondary anti-rabbit-Alexa 488 and anti-rat-Alexa 546 antibodies diluted to 1 : 500 in PBS and 0.1% BSA for 1 h at 4°C. The secondary antibodies were used in the absence of primary antibodies as negative controls. After labelling, cells were mounted on slides using Vectashield with DAPI (Vector, Burlingame, CA, USA) and examined on a TCS-SP5 AOBS confocal microscope (Leica Microsystems, Germany). Images were analyzed using the ImageJ software. Each cell micrograph was analyzed using ImageJ software (Rasband, W.S., ImageJ, US National Institutes of Health, Bethesda, Maryland, USA, http://imagej.nih.gov/ij/, 1997–2014). The region of interest (ROI) was delimited in each cell and the respective mean fluorescence intensity was measured based on the pixel intensity. 10 cells per image were evaluated and the fluorescent values were used for statistical analysis. Data were also displayed in an orthogonal slice view that shows the raw pixel intensity values found mutually in each of three perpendicular planes.

### 2.9. Western Blotting

Macrophages adhered to 6-well plates were washed with sterile PBS and lysed with 200 *μ*L RIPA buffer (50 mM Tris-HCl, pH 7.4, containing 1% Nonidet P40, 0.25% sodium deoxycholate, 150 mM NaCl, 1 mM EDTA, 1 mM PMSF, 1 mM sodium orthovanadate, 1 mM NaF, 10 *μ*g/mL aprotinin, and 10 *μ*g/mL leupeptin). Cell lysates were incubated at 4°C for 30 min and centrifuged at 8,100 ×g, for 10 min, at 4°C; the pellets were discarded and the supernatant had their protein content determined by the Lowry method [33]. Samples were run on 10% SDS–PAGE gels (15 *μ*g protein/lane) and then transferred to nitrocellulose membranes using a semidry transfer system. Membranes were blocked for 1 h in Tris-buffered saline (TBS) with 2% nonfat milk and then incubated overnight with rabbit anti-P2X7 receptors (APR-004; Alomone Labs, Israel; 1 : 200; Peptide (C)KIRKEFPKTQGQYSGFKYPY, corresponding to amino acid residues 576–595 of P2X7 receptor intracellular, C-terminus) or mouse monoclonal anti-*β*-actin (1 : 20.000) primary antibodies. After 3 washes in TBS-Tween, membranes were incubated with HRP-conjugated anti-rabbit or anti-mouse secondary antibody (1 : 1.000) for 1 h and developed using enhanced chemiluminescence (ECL). Ponceau staining and labeling with a monoclonal anti-*β*-actin antibody were used as internal controls for protein loading. Densitometry of protein bands was performed using the ImageJ software, and the values for relative amounts of protein were normalized to the *β*-actin loading control.

### 2.10. Quantitative Real Time PCR (qRT-PCR)

Peritoneal macrophages from uninfected* Swiss* mice (treated with 5 ng/mL TGF-*β*1 or with vehicle, for 24 h) or from* S. mansoni*-infected mice were used to isolate total RNA using the TRIzol reagent (Life Technologies), according to the manufacturer's instructions. Total RNA was quantified using an ND-1000 spectrophotometer (NanoDrop), and cDNA was synthesized from 500 ng of total RNA using the high capacity cDNA reverse transcription kit with RNase inhibitor (Invitrogen). The SYBR Select Master Mix (Applied Biosystems) was used for qRT-PCR, to detect double-stranded DNA synthesis. Reactions were carried out in a final volume of 10 *μ*L, using 2 *μ*L of diluted cDNA (1 : 10) and 300 nM of each of the reverse and forward primers. The following primers were used for qRT-PCR: for* P2rx7*, 5′ AATCGGTGTGTTTCCTTTGG 3′ (forward) and 5′ CCGGGTGACTTTGTTTGTCT 3′ (reverse); for* Actb*, 5′ TATGCCAACACAGTGCTGTCTGG 3′ (forward) and 5′ TACTCCTGCTTGCTGATCCACAT 3′ (reverse); and for* Gapdh*, 5′ GGTCATCCCAGAGCTGAACG 3′ (forward) and 5′ TTGCTGTTGAAGTCGCAGGA 3′ (reverse).

Reactions were performed in a 7500 Fast Real-Time System (Applied Biosystems), using the following PCR conditions: 5 min at 95°C, followed by 40 cycles of 15 s at 95°C, 35 s at 60°C, and 15 s at 72°C. At the end of cycling protocol, a melting-curve analysis (with fluorescence measurements from 60 to 99°C) was performed. Relative expression levels were determined using the Sequence Detection Software v.2.0.5 (Applied Biosystems), and the efficiency per sample was calculated using the LinRegPCR 11.0 software (manufacturer).*β*-*Actin* and* Gapdh* were used as internal controls to calculate relative* P2rx7 *mRNA levels, by the comparative threshold cycle (ΔΔC_T_) method with efficiency correction, using the mean qRT-PCR efficiency for each amplicon, as previously described [34, 35].

### 2.11. Statistical Analysis

Data were expressed as mean and SEM. Differences between two or more groups were analyzed by Student's *t*-test or one-way analysis of variance (ANOVA) followed by post hoc Newman-Keuls test, respectively, considering *P* < 0.05.

## 3. Results

### 3.1. P2X7 Receptor Function Is Reduced in Peritoneal Macrophages during Schistosomiasis

**Figure 1:**
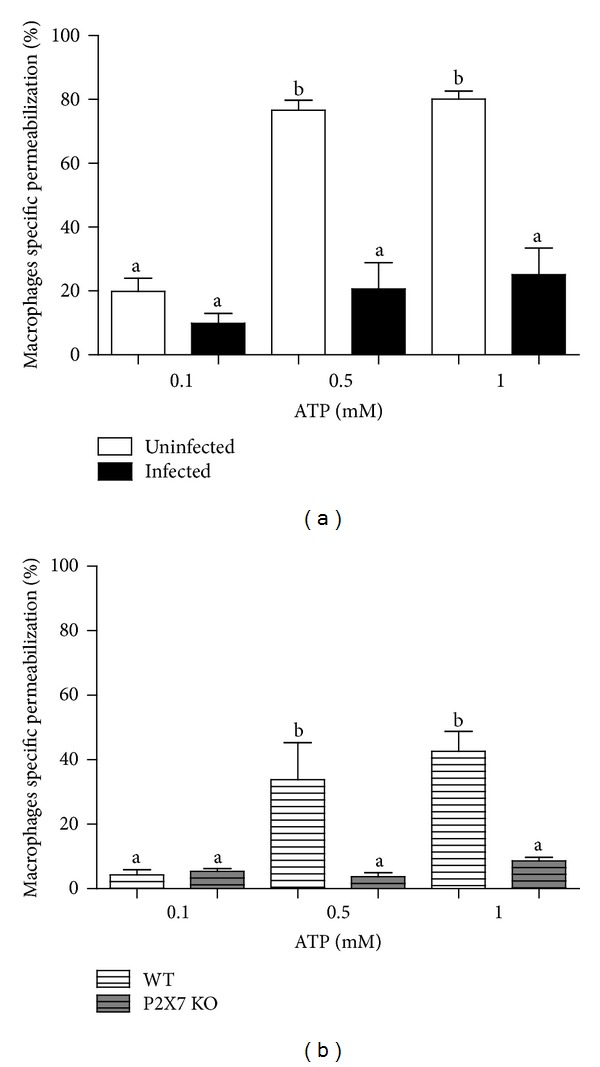
Evaluation of ATP-induced permeabilization in peritoneal macrophages, based on ethidium bromide (EB) uptake. (a) Peritoneal macrophages from* S. mansoni*-infected* Swiss* mice (black bars) were less sensitive to ATP-induced permeabilization than those from uninfected mice (white bars). (b) Peritoneal macrophages from C57BL/6 wild type mice (hatched white bars) respond to ATP-induced permeabilization unlike those from P2X7R KO mice (hatched gray bars). ATP-induced permeabilization values represent the % of EB uptake above basal levels by F4/80 positive cells and were expressed as mean and SEM. *N* = 12 (*Swiss*) and *n* = 6 − 9 (C57BL/6) replicates using different animals. (a) and (b) differ from each other with *P* < 0.001 (one-way ANOVA followed by post hoc Newman-Keuls test).

We used several approaches to examine the impact of the chronic inflammation triggered by* S. mansoni* infection on macrophage P2X7 receptor function. Activation of P2X7 receptors by ATP opens plasma membrane pores that allow molecules greater than 900 kDa (such as the fluorescent dye EB) to enter the cells [17, 36]. Thus, we used ATP-induced cell permeabilization (as evidenced by staining with EB) as a tool to compare the P2X7 receptor activation in peritoneal macrophage from infected and uninfected* Swiss* mice. Incubations with 0.1–1 mM ATP induced cell permeabilization in macrophages from uninfected mice; however, this effect was reduced in macrophages from infected mice (Figure 1(a)). ATP also induced permeabilization in macrophages from C57BL/6 wild type mice but, as expected, not in macrophages from P2X7 KO mice (Figure 1(b)).

**Figure 2:**
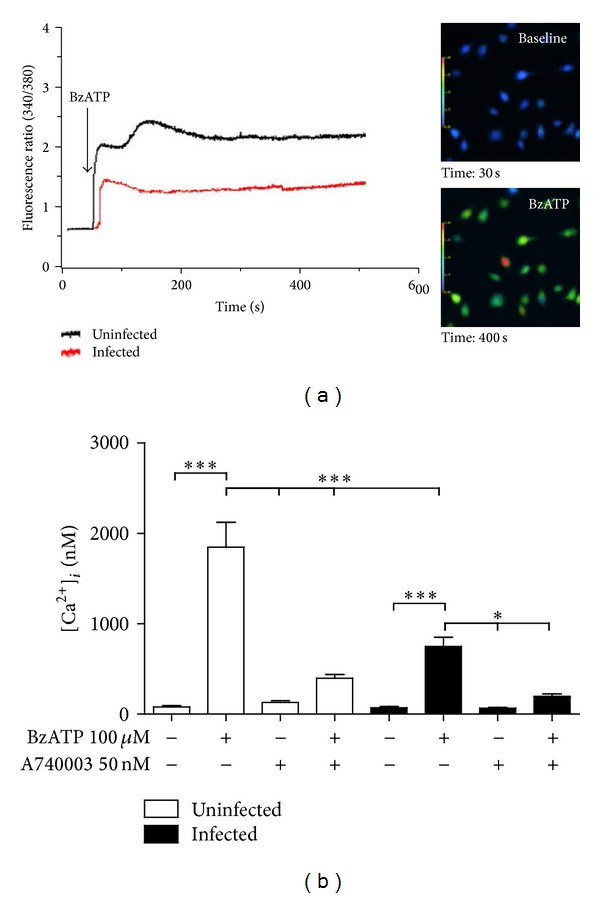
Intracellular Ca^2+^ measurement (using Fura-2-AM) in macrophages subjected to treatment with the P2X7 agonist BzATP and/or with the antagonist A740003. (a) Changes in intracellular Ca^2+^ levels in macrophages from control (black line) and* S. mansoni*-infected (red line) mice. Inset: representative images from macrophages (control) before (baseline) and after treatment with 100 *μ*M BzATP. (b) Changes in intracellular Ca^2+^ levels ([Ca^2+^]_*i*_) in macrophages from control (white bars) or* S. mansoni-*infected (black bars) mice. Three plates were used for each condition/animal, 10 cells/plate were chosen randomly for imaging, and cells were obtained from three animals. Data were expressed as mean and SEM. *P < 0.05; ***P < 0.001 (one-way ANOVA followed by post hoc Newman-Keuls test).

We also used the potent P2X7 agonist BzATP to verify if P2X7 receptor activation increased the cytosolic Ca^2+^ concentration in macrophages from infected and uninfected* Swiss* mice, since ATP-induced Ca^2+^ influx is a hallmark of P2X7 activation [36]. The basal levels of cytosolic Ca^2+^ (before BzATP addition) were similar in both groups (Figure 2(a)). After treatment with 100 *μ*M BzATP, macrophages from uninfected mice showed a typical P2X7 receptors activation profile at 37°C, characterized by a pronounced biphasic increase in intracellular Ca^2+^ levels (Figure 2(a)) [36]. However, the P2X7 receptor-mediated Ca^2+^ influx was considerably less pronounced in macrophages from infected mice when compared to that observed in macrophages from uninfected mice (Figures 2(a) and 2(b)). We also assessed changes in intracellular Ca^2+^ in the presence of the selective P2X7 receptor antagonist A740003. Treatment with 50 nM A740003 did not alter basal intracellular Ca^2+^ levels, but it had a strong negative effect on the BzATP-induced Ca^2+^ influx, and this effect was similar in macrophages from both groups. These results confirmed that the increase in intracellular Ca^2+^ levels after addition of BzATP was due to P2X7 receptor activation. These data are in agreement with the reduction in ATP-induced permeabilization observed in macrophages from infected mice (Figure 1(a)) and suggest that P2X7 receptor signaling is downregulated during schistosomiasis.

### 3.2. *S. Mansoni*-Infected Mice Have Higher Levels of Peritoneal TGF-*β*1, Associated with Decreased P2X7-Dependent Macrophage Permeabilization

**Figure 3:**
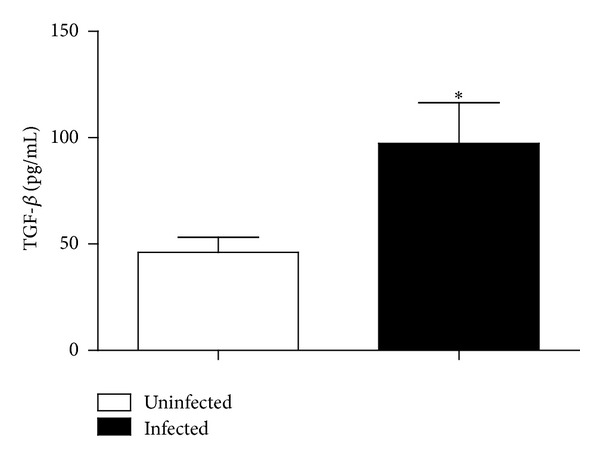
Peritoneal TGF-*β*1 levels in uninfected (white) and* S. mansoni*-infected mice (black). Data were expressed as mean and SEM. *n* = 6 (uninfected) or 4 (infected) mice. *P < 0.05 (Student's *t*-test).

The anti-inflammatory cytokine TGF-*β*1 prevents P2X7 receptor upregulation in monocytes [37]. Moreover, in the chronic phase of schistosomiasis, there is a gradual increase of serum Th2 cytokines [21]. Indeed, we observed a significant increase in TGF-*β*1 levels in the peritoneal cavity of mice infected with* S. mansoni* (Figure 3).

**Figure 4:**
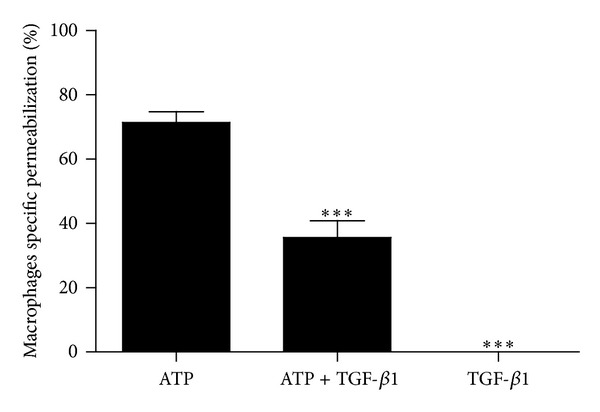
ATP-induced permeabilization assays (based on ethidium bromide uptake) by peritoneal F4/80 positive macrophages of uninfected mice treated with TGF-*β*1. Cells treated with 5 ng/mL TGF-*β*1 or vehicle for 24 h were stimulated with 1 mM ATP. ATP-induced permeabilization values represent the % of EB uptake above basal levels and were expressed as mean and SEM. *n* = 8–21 replicates using 6 animals (***P < 0.001 versus ATP, one-way ANOVA followed by post hoc Newman-Keuls test).

To investigate if TGF-*β*1 could modulate P2X7 receptor function in peritoneal macrophages similar to that described for monocytes [37], we harvested peritoneal macrophages from uninfected animals and treated these cells with 5 ng/mL TGF-*β*1 for 24 h before assessing their sensitivity to ATP-induced permeabilization (using EB as a tracer). In the absence of ATP, TGF-*β*1 treatment did not induce cell permeabilization; however, TGF-*β*1 reduced the permeabilizing effect of 1 mM ATP by approximately 50%, when compared to controls treated with ATP only (Figure 4). We also performed the permeabilization after 48 h of TGF-*β* treatment and we observed that, even after 48 h, the P2X7-induced permeabilization was also reduced (data not shown). The analysis of the concentration-response curve using different concentrations of TGF-*β* showed that only the concentrations 5 ng/mL and 10 ng/mL were able to reduce the permeabilization intensity in macrophages (data not shown).

### 3.3. P2X7 Receptor Protein and mRNA Levels Do Not Change after Infection with* S. Mansoni* or Treatment with TGF-*β*1

**Figure 5:**
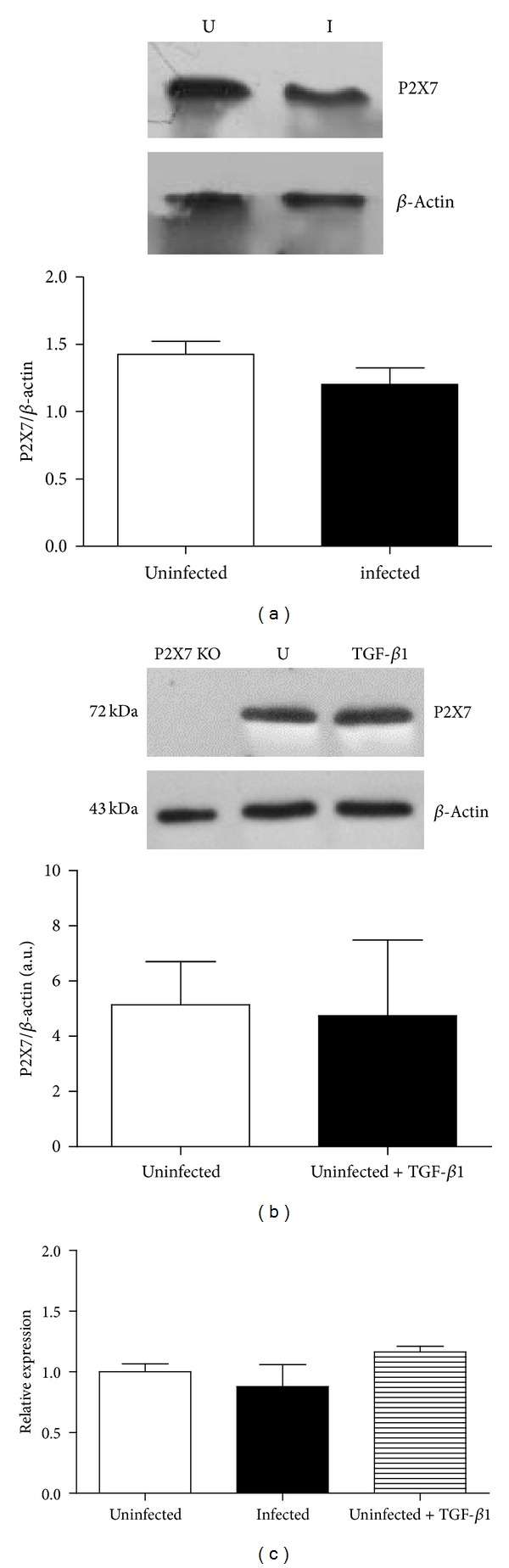
P2X7 receptors expression in peritoneal macrophages. (a) Macrophages from uninfected (white bar) and* S. mansoni*-infected (black bar) mice express similar levels of P2X7 protein. *n* = 9 replicates from three different animals, for each group. The images above are representative Western blots (U = uninfected and I = infected). (b) P2X7 receptors expression in peritoneal macrophages from control mice (white bar, untreated) or TGF-*β*1-treated cells (5 ng/mL, 24 h; black bar) are also similar. The images above are representative Western blots (U = uninfected; TGF-*β*1= TGF-*β*1-treated macrophages). Lysates from P2X7 KO macrophages were used as negative controls for P2X7 protein expression. Cells lysates were obtained from three animals for each group. (c) Quantitative RT-PCR (qRT-PCR) analysis of P2X7 receptor mRNA levels in peritoneal macrophages. Similar levels of P2X7 mRNA were found in uninfected (white bar),* S. mansoni*-infected mice (black bar), and TGF-*β*1-treated (5 ng/mL, 24 h; hatched bar) mouse macrophages. *N* = 3–5 samples per group (using different animals).

To evaluate whether the reduction in P2X7 receptors function observed in schistosomiasis and in TGF-*β*1-treated cells was due, at least in part, to changes in P2X7 receptor expression, we quantified both P2X7 protein expression and the transcription from the* P2rx7* locus by Western blotting and qRT-PCR, respectively. We detected similar levels of P2X7 protein and mRNA in macrophages from uninfected and infected groups (Figures 5(a) and 5(c)). Moreover, the levels of P2X7 protein and mRNA in macrophages from uninfected mice were not altered by treatment with 5 ng/mL TGF-*β*1 for 24 h (Figures 5(b) and 5(c)).

### 3.4. Schistosomiasis and Treatment with TGF-*β*1 Reduce Cell Surface P2X7 Levels in Peritoneal F4/80 Positive Macrophages

P2X7 receptors are targets of posttranslational modifications that regulate their insertion into plasma membrane [38], and the proinflammatory effects of P2X7 receptors (including IL-1*β* release and NO and ROS production) depend on receptor localization at the cell surface. Moreover, prolonged exposure of macrophages to ATP may induce P2X7 receptors removal from the cell surface by internalization [39, 40].

**Figure 6:**
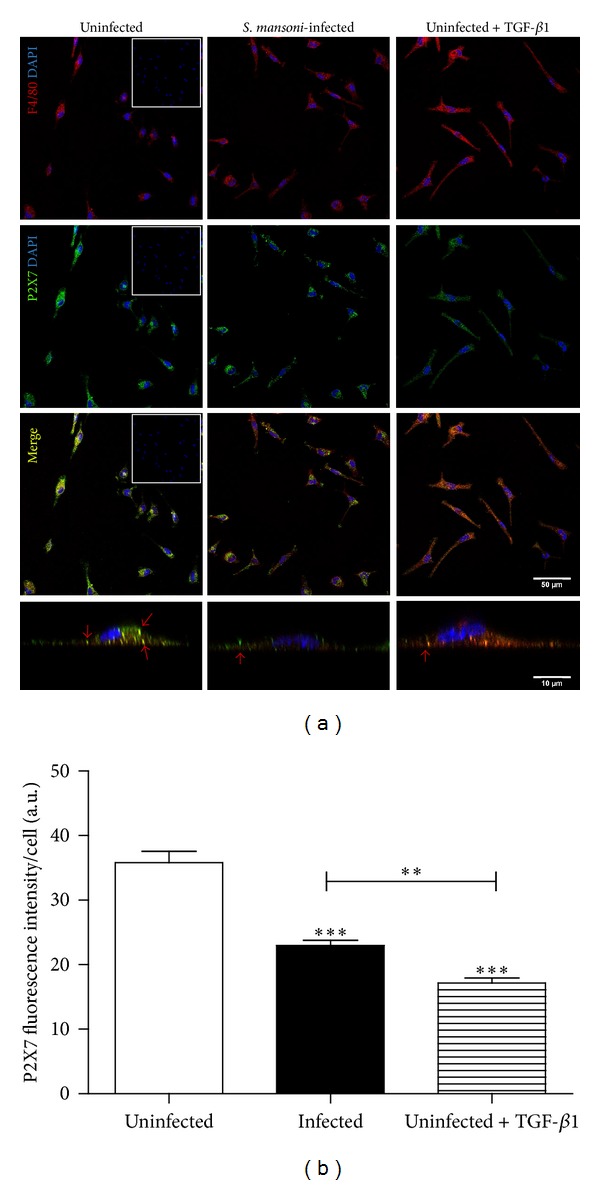
Confocal microscopy analysis of P2X7 receptor expression on the cell surface of macrophages. Cells were labelled with antibodies recognizing P2X7 receptors (green) and F4/80+ (red) as well as with DAPI (blue). (a) Representative images showing macrophages from uninfected (left column),* S. mansoni*-infected (middle column), and TGF-*β*1-treated (right column; 5 ng/mL, for 24 h) mice. The bottom row shows orthogonal slices of the representative labelled cells, with red arrows marking P2X7 expression on the plasma membrane. (b) Quantitative analysis of confocal microscopy data. The mean fluorescence intensity, showing cell surface P2X7 receptor expression in peritoneal macrophages from uninfected mice (white bar),* S. mansoni*-infected (black bar), and TGF-*β*1-treated (hatched bar; 5 ng/mL, for 24 h) mice, was obtained from pixels intensity using ImageJ software (see methods). Inset: negative controls (macrophages incubated with secondary antibodies only). *n* = 6 replicates from 3 animals for each condition (***P < 0.001 versus control, *P* < 0.01 TGF-*β*1 versus infected group; one-way ANOVA followed by post hoc Newman-Keuls test).

Since the reduction in P2X7 function during* S. mansoni* infection was not due to decreases in protein or mRNA levels, we hypothesized that changes in receptor structure or localization occurred during infection and downregulated receptor function, similar to that described for other P2X receptors [41]. To test this hypothesis, we analyzed P2X7 receptor localization in nonpermeabilized F4/80 (a common surface marker of mature macrophages) positive macrophages from uninfected and* S. mansoni*-infected mice using an antibody that recognizes an extracellular P2X7 epitope. Confocal microscopy analysis of anti-P2X7 labeled cells suggested that the levels of cell surface P2X7 receptors were reduced in macrophages from infected mice (Figures 6(a) and 6(b)). Interestingly, there was also a reduction in cell surface P2X7 in macrophages from uninfected mice treated with TGF-*β*1 (Figures 6(a) and 6(b)). Relative quantification of the immunofluorescence data showed that there was a 1.6-fold decrease (approximately) in the intensity of cell surface P2X7 fluorescence in macrophages from infected mice, when compared to that observed in uninfected control mice (Figure 6(b)).

### 3.5. Reduced Survival of P2X7 KO Mice Infected with* S. Mansoni*

**Figure 7:**
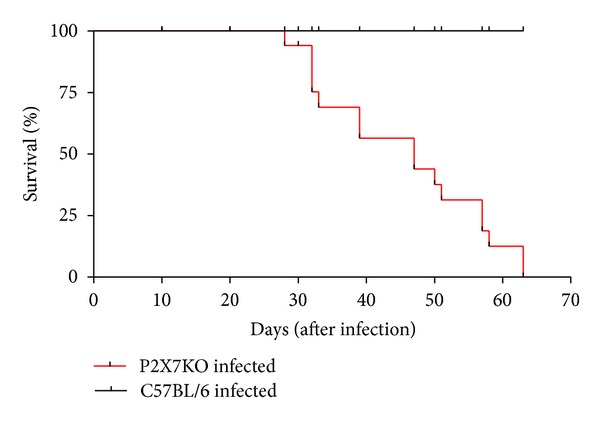
Survival curve of C57BL/6 wild type or P2X7 KO infected with approximately 80 cercariae of* S. mansoni*. (P2X7 KO: *n* = 9 females and 7 males; C57BL/6 wild type: 4 females and 10 males). ***P < 0.0001 using the Mantel-Cox log-rank test.

Next, to evaluate the participation of P2X7 receptors during the disease, we infected C57BL/6 wild type and P2X7 KO mice and followed the progression of infection. The survival curves showed that mortality in P2X7 KO infected mice started at 28 dpi and reached 100% at 60 dpi. In contrast, no death was observed in C57BL/6 wild type mice (Figure 7).

## 4. Discussion

During schistosomiasis, macrophages may display classical or activated phenotypes. The latter phenotype is present mainly in macrophages surrounding eggs within liver granulomas and was recently shown to be important in the control of tissue fibrosis [42]. Considering that schistosomiasis is a chronic inflammatory disease and that macrophages are involved in schistosomiasis pathogenesis [24], we sought to evaluate the function of P2X7 receptors in macrophages from mice infected with* S. mansoni*, since these receptors play an important role in inflammatory processes.

Here, we used a combination of ATP-induced permeabilization and intracellular Ca^2+^ measurement assays to show that, during the chronic phase of schistosomiasis, there is a reduction in the ATP-dependent P2X7 receptor function in macrophages (Figures 1 and 2), similar to that observed in mesenteric endothelial cells using the same experimental model [43].

While the function and expression of macrophage P2X7 receptors are positively regulated by proinflammatory cytokines such as IFN-*γ* [44, 45], Gadeock and coworkers [37] showed that the upregulation of P2X7 receptors in THP-1 monocytes is negatively regulated by TGF-*β*. On the other hand, peripheral blood mononuclear cells (PBMCs) from* S. mansoni*-infected mice produce high levels of TGF-*β* [27], and this cytokine is important to limit liver inflammation and favor host survival [27, 28]. Overall, these data are in agreement with our results also showing that the levels of peritoneal TGF-*β*1 are increased in mice infected with* S. mansoni *(Figure 3) and that TGF-*β*1, in a concentration close to the ones observed in the serum of chronic patients [46], reduces by approximately 50% the P2X7-dependent macrophage permeabilization triggered by ATP (Figure 4), mimicking the attenuated P2X7 response observed during* S. mansoni* infection (Figure 1(a)). Taken together, these results support our hypothesis that the downregulation of P2X7 function observed in schistosomiasis is a result of the increase in TGF-*β*1 levels during infection. Regarding TGF-*β* levels, despite the difference between the* in vitro* treatment concentration (5 ng/mL) and the* in vivo* peritoneal measurement (100 pg/mL), we must consider that we injected 1 mL of PBS in the peritoneal cavity of infected animals before removing the body fluid for cytokine determination. Therefore the samples were diluted, and, as a consequence, peritoneal TGF-*β* levels* in vivo* may be higher than the estimation* in vitro*. Moreover, previous data showed that individuals chronically infected with schistosomiasis have TGF-*β* serum levels around 20 ng/mL [46] and the serum level estimated in* S. mansoni*-infected mice (19.98 ± 2.37 ng/mL) is compatible with the value observed in patients [47]. Therefore we conclude that the TGF-*β* concentration used in present work is compatible with the disease, and the peritoneal TGF-*β* concentration in the infected animals is probably higher than estimated. Finally, TGF-*β* in the ng/mL range of concentration is able to modulate P2X7R function.

Western blotting and qRT-PCR data (using whole cell lysates) excluded the possibility that the reduction in P2X7 receptor function during* S. mansoni* infection or after treatment with TGF-*β*1 resulted from decreased levels of P2X7 protein or mRNA. In fact, immunofluorescence microscopy analysis of nonpermeabilized macrophages expressing F4/80 on their cell surface revealed that there was a lower density of surface P2X7 receptors in cells from infected mice compared to those from uninfected, control animals (Figure 6). Since total P2X7 protein levels were similar in both groups, we propose that there is reduction of P2X7 receptor in plasma membrane of macrophages from infected mice. The disappearance of immunoreactivity from plasma membrane could be related to some conformational changes of the receptor, an interaction with other proteins masking the extracellular epitope recognized by the antibody or the P2X7 receptor internalization. However, TGF-*β* treatment* in vitro* mimicked both the reduced cell surface receptor expression and function observed in macrophages obtained from infected animals. Consequently, a plausible explanation for the reduction in P2X7 function (without corresponding decreases in total protein or mRNA levels) during chronic schistosomiasis could be related to a reduced cell surface expression of P2X7 receptor.

Despite the work of Gadeock et al. [37], data investigating the link between TGF-*β*1 and P2X7 receptors function and/or expression are still missing. Since control macrophage treatment with TGF-*β*1 mimicked the profile of cell surface P2X7 receptors expression and function observed in live macrophages obtained from infected mice, it is supposed that TGF-*β*1 could be involved in the P2X7 receptors reduced function observed in the disease. To the best of our knowledge, this is the first report that shows a direct effect of TGF-*β*1 on macrophage P2X7 receptor function. However, we do not exclude that other cytokines could contribute to the P2X7 reduced function in macrophages from* S. mansoni*-infected mice, since in the later phase of the disease beyond the increase of TGF-*β* levels there is also the increase of IL-4, IL-5, IL-10, and IL-13.. Moreover, it was shown that IL-4 and IL-10 also inhibit EB uptake in rat alveolar macrophage [48]. Furthermore, previous data showed that TGF-*β*, IL-10, and IL-4 inhibit macrophage cytotoxicity which could be an important strategy used by* S. mansoni* to evade macrophage-mediated immune destruction [49].

Patients with schistosomiasis may be more susceptible to secondary infections, and prior infection with* Schistosoma* also increases the severity of secondary infections with* Leishmania, Toxoplasma gondii,* and* Salmonella* [50]. In addition, a previous report showed that macrophages from chronically infected mice have reduced phagocytic activity [51]; however, little is known about the mechanisms underlying this phenomenon. Recently, Wiley and Gu [52] showed that P2X7 receptors on the surface of monocytes/macrophages may act as scavenger receptors for bacteria in the absence of ATP. Therefore, the reduction of P2X7 receptor function in* S. mansoni*-infected animals could limit bacterial (and possibly protozoal) phagocytosis during schistosomiasis. This phenomenon might explain the increased susceptibility of patients infected with* S. mansoni* to secondary infections with other parasites and also the higher severity of secondary infections in these patients.

Kusner and Adams [53] showed that ATP kills* Mycobacterium tuberculosis* in human macrophages and that this effect depends on P2X7 receptor-mediated PLD activation. This mechanism is also important for the ATP-mediated killing of* Chlamydia trachomatis *in murine peritoneal macrophages [13]. Moreover, our group showed that the acute infection of macrophages with* Chlamydia psittaci *reduces P2X7 receptor-mediated cell permeabilization and Ca^2+^ influx, thus inhibiting macrophage apoptosis [54]. Considering that* C. psittaci *are obligatory intracellular parasites, this phenomenon could be an attempt to limit immunological responses by reducing ATP-mediated apoptosis and, consequently, favoring parasite survival. An important new aspect of P2X7 receptors signaling highlighted in the present work is that the function of macrophage P2X7 receptors may also be modulated by the extracellular parasite* S. mansoni*.

Altogether, our data suggest that, during schistosomiasis, the function of macrophage P2X7 receptors is reduced and that TGF-*β*1 plays a key putative role in the downregulation of receptor signaling. We hypothesize that macrophage purinergic receptors are differentially modulated during disease progression and that this phenomenon has a key role in the immune response against* S. mansoni*. In this context, our data show that P2X7 receptor knockout animals (P2X7 KO) are much more susceptible to death during* S. mansoni* infection than WT mice highlighting this P2 purinergic receptor and reinforcing the necessity of future studies exploring in deep its importance in* S. mansoni*-induced disease. Given that purinergic signaling is also found in* S. mansoni*, understanding the interplay between host and parasite purinergic signaling pathways is now important to clarify if future therapeutic approaches targeting these signaling pathways would be useful against schistosomiasis.

## 5. Conclusions

Altogether, our data show that* S. mansoni* infection reduced P2X7 function in peritoneal macrophages during the chronic phase of the disease. Furthermore, the peritoneal cavity of infected mice had increased levels of TGF-*β*1, and this cytokine reduced P2X7 receptor function in macrophages from uninfected mice. Thus, immunomodulation by TGF-*β*1 could limit P2X7-dependent inflammatory effects in macrophages from* S. mansoni* patients and may provide an explanation for the increased susceptibility of these patients to infections by others pathogens.
